# Ecological setup, ploidy diversity, and reproductive biology of *Paspalum modestum*, a promising wetland forage grass from South America

**DOI:** 10.1590/1678-4685-GMB-2019-0101

**Published:** 2020-02-21

**Authors:** Piyal Karunarathne, Cristian Feduzka, Hojsgaard Diego

**Affiliations:** 1 University of Goettingen, Albrecht-von-Haller Institute for Plant Sciences, Department of Systematics, Biodiversity and Evolution of Plants (with Herbarium), Goettingen, Germany.; 2 University of Goettingen, Georg-August University School of Science, Germany.; 3 Facultad de Ciencias Agrarias, Universidad Nacional del Nordeste (FCA-UNNE), Instituto de Botánica del Nordeste (IBONE), Corrientes, Argentina.

**Keywords:** Apomixis, ecological niche, plant breeding, polyploidy, sexuality

## Abstract

With ever-rising demand for food, forage breeding for intensification of cattle production is also taking a leap. In South America, cattle production systems are displaced to marginal areas poorly exploited with cultivated pastures yet with high potential for growing stocking rates. This places the need for using native genetic resources to breed locally adapted plant genotypes that benefits from better forage quality, yield, and lesser threat to the local biodiversity. *Paspalum modestum* Mez is a grass species that produces quality forage and grows in marginal areas like estuaries and floodplains, suitable for introduction in breeding programs. In this study we characterize the species' reproductive biology and ecological preferences needed beforehand any improvement. *P. modestum* plants found in nature are commonly diploids, rarely triploids, and tetraploids. Chromosome associations during meiosis in polyploids indicate they are autopolyploids. While diploids are sexual self-sterile, analyses of embryology, gamete fertility and experimental crossings show tetraploids are self-compatible facultative apomicts, highly fertile and have a high proportion of sexuality compared to other apomictic species. Ecological niche analysis and species distribution modelling show mean annual temperature and precipitation as main ecological drivers and a wide geographical area of climatic suitability where *P. modestum* can grow and be exploited as a forage grass. Our study points to *P. modestum* as a native plant resource appropriate for breeding waterlogging tolerant ecotypes and genotypes of high biomass production adapted to low flow areas in the Subtropics of Brazil, Paraguay, Uruguay and Argentina.

## Introduction

At present, increasing agricultural productivity and land-use sustainability is a primary aim of most national governments and transnational organizations. Clearance for agriculture and/or cattle ranching is the dominant land-cover change in South America (Hansen *et al.*, 2013). The expansion of the agricultural frontier seems to be poorly selective (e.g., Volante *et al.*, 2016) and therefore imposes a threat to biodiversity and environmental sustainability through landscape fragmentation, changes in productivity and carbon balance (Gasparri *et al.*, 2008), and dissipation of primary production (Verón *et al.*, 2012). Even though elements influencing the dynamics of land-use change are complex, multi-tiered approaches including policies to reduce deforestation, diversified agricultural production, ranching intensification, and management innovations can provide opportunities for developing environmental sustainable landscapes (Galford *et al.*, 2013). Within this frame, technological factors including the availability of genetic materials are particularly relevant.

In Argentina, Paraguay, and Brazil, cattle production systems have been subsequently displaced by agriculture into marginal areas, supporting lower livestock density per hectare. In addition, projections point toward an increase in grazing pressure and escalation of livestock production in humid and semi-humid grazing systems around the world (Thornton, 2010). Therefore, the search and use of plant materials locally adapted into breeding programs can provide better forage quality, increased forage yield, grazing options compared to introduced species, and a lesser threat to the local biodiversity.

Marginal cropland areas in South America are mainly represented by highlands or lowlands, swamplands, and wetlands which comprise high biodiversity spots, are crucial providers of numerous ecosystem services, and therefore foci for conservation (Wittmann *et al.*, 2015). A wide variety of fodder species grow associated to wetlands and a few of them show promising forage potential. *Paspalum modestum* Mez is one such species, suitable for introducing in breeding programs aimed at selecting varieties with increased herbage mass.

Forage improvement in *Paspalum* spp., as well as in other grass genera used in tropical, subtropical, and temperate grasslands around the world, exploits a particular reproduction strategy called apomixis, commonly found among polyploid plants of such genera (Quarin 1992; Ortiz *et al.*, 2013). While normal sexual reproduction refurbishes genetic information during gamete and offspring formation, apomixis avoids genetic segregation and thus produce uniform progeny (e.g., Quero Carrillo *et al.*, 2010; Acuña *et al.*, 2011).

*Paspalum modestum* belongs to the Plicatula group, one out of about 30 infrageneric groupings of species within the genus *Paspalum*, defined through shared morphological and ecological similarities (Chase, 1929; Parodi and Nicora, 1966; Barreto, 1974; Zuloaga and Morrone, 2005). Plicatula includes 4 out of 10 *Paspalum* species cultivated around the world as forage (i.e., *P. guenoarum* Arechav., *P. glaucescens* Hack., *P. plicatulum* Michx., *P. atratum* Swallen) (Burton, 1940; Ramirez, 1954; Barreto, 1974; Judziewicz, 1990; Espinoza *et al.*, 2001; Evers and Burson, 2004). The possibility to create superior genotypes able to transfer the selected features unchanged to the, consequently uniform, offspring in *P. modestum* was first limited by the lack of polyploid apomictic plants. Until a few years ago, only diploid (2*n* = 2*x* = 20) self-sterile sexual individual plants were available (Quarin and Hanna, 1980). For this reason, and driven by the forage quality and particular ecology of the species, diploid cytotypes of *P. modestum* were introgressed to tetraploid cytotypes of *P. notatum* Flüggé, a well-known forage species (Quarin, 1983). However, the obtained hybrids were triploid (2*n* = 3*x* = 30) and fully sterile, likely due to the odd ploidy condition. Further efforts attempting to duplicate the chromosome number of the hybrids to escape sterility have been unsuccessful, and *P. modestum* was therefore discarded from the *P. notatum* breeding scheme (Quarin, 1983).

In the last few years, the identification of *P. modestum* tetraploid cytotypes (2*n* = 4*x* = 40) in Brazil (Pozzobon and Valls, 2003) and Argentina (Hojsgaard *et al.*, 2009) opened up the possibility of incorporating the species in current breeding program. According to Reis (2007), forage evaluations of diploid and tetraploid plant materials of the species show that the polyploids are more productive. In spite of the fact that the evaluated materials exhibited low tolerance to drought and frost, they showed a better recovering capacity and high forage production during the rainy periods of the hot season when compared to other *Paspalum* species (Reis, 2007).

*Paspalum modestum* is a natural pasture growing in estuaries, borders of shallow waters and floodplains around the Subtropics in southern Brazil, Paraguay, Uruguay, and northern Argentina (Burkart, 1969; Zuloaga and Morrone, 2005). The apparent ecological aptitudes of the species together with its forage aptitudes can turn tetraploid cytotypes of *P. modestum* to a native, local resource with a high potential for forage production, either by creating breed materials or by introgressing *P. modestum* features into other forage species within *Paspalum*. In geographic areas with low slopes and abundant water regimes, *P. modestum* can provide good hay where it is adapted to grow in shallow zones of permanent ponds and regularly flooded inlets.

In the present work, we aim at determining the ecological preferences of the species in order to estimate the potential range and areas of cultivation, and at characterizing the reproductive mode and fertility of tetraploid plant materials suitable to be incorporated in breeding programs. Therefore, we (i) use occurrence data in species distribution modellings, (ii) determine ploidy levels of different plant materials from new collection sites, (iii) analyze the microsporogenesis, microgametogenesis and pollen viability of tetraploid cytotypes, (iv) analyze the megasporogenesis, megagametogenesis and fertility of tetraploids, and (v) use progeny tests to further characterize the reproductive mode of tetraploids and the genetic uniformity of offspring produced under different breeding conditions.

## Material and Methods

### Plant material and species occurrence

*Paspalum modestum* plants were collected in different collection trips within Argentina (Table 1) and are cultivated at the Facultad de Ciencias Agrarias, Universidad Nacional del Nordeste in Corrientes. Vouchers were deposited at the Herbarium of the Botanical Institute of the Northeast (CTES) and the Herbarium of the Botanical Institute Darwinion (SI). Occurrence data was obtained from personal collections, GBIF database (https://www.gbif.org/), Documenta Flora Australis database (IRIS database; http://www2.darwin.edu.ar/iris/), FLORA ARGENTINA (www.floraargentina.edu.ar), and from the Herbaria CTES (http://ibone.unne.edu.ar/en) and SI (http://www2.darwin.edu.ar/). For a few cases from old collections with detailed information on localities, localities were georeferenced using the Point method.

**Table 1:** Collection sites and ploidy levels of plant materials.

Locality	Plants	2*n*
**Corrientes province**		
Dpt. Lavalle, Santa Lucia	2	20
Dpt. San Roque, San Roque ^1^	4	20
Dpt. Itatí, Ramada Paso ^2^	4	20
Dpt. General Paz, Itá Ibaté ^3^	7	20
	1	**40**
Dpt. San Cosme, San Cosme	2	20
Dpt. Ituzaingó, Ituzaingó ^4^	1	20
	6	**40**
**Chaco province**		
Dpt. Bermejo, La Leonesa	1	30

Vouchers deposited at CTES and SI: 1) *Hojs392*, **2)***Hojs391*, **3**) *Hojs333*, **4)***Hojs395*.

### Ploidy analysis and meiosis

Mitotic chromosome counts from root tips were used to determine the ploidy level of plant materials. Root tips were collected from cultivated individuals, treated in a saturated aqueous solution of α-bromonaphthalene for 2.5 h, hydrolysed in a 1N HCl solution for 10 min at 60 °C, and finally stained with Feulgen’s reagent for approximately 30 min before the tips were squashed in a drop of 2% acetic orcein.

Meiotic chromosomes from young spikelets were used to analyse chromosomal configurations and gamete formation. Inflorescences before emerging from the flag leaf sheath were fixed in a 3: 1 solution of absolute ethanol: glacial acetic acid and stored in 70% ethanol at 5 °C. Anthers were dissected from single spikelets and pollen mother cells (PMC) were stained with 2% acetocarmine. Slides were made permanent with Venetian turpentine. Meiotic stages and chromosome associations were studied using a bright field Leica Photostar II microscope (Leica Microsystems, Wetzlar, Germany).

### Embryology

For the analyses of megasporogenesis and megagametogenesis, young inflorescences and spikelets at anthesis were collected and fixed in FAA (70% ethanol, 37% formaldehyde, glacial acetic acid, 18:2:1). Single spikelets were dissected under a Wild M5A stereoscope and subjected to two different protocols. For the sectioning and staining protocol, spikelets were dehydrated in a series of tertiary butyl alcohols, embedded in paraffin, sectioned at 12 μm and stained using Safranin and Fast-green. Sections were analysed in a Leica DM2500 bright field microscope (Leica Microsystems) and images were taken with a DFC320 camera. For the clearing protocol, dissected spikelets were bleached using an aqueous solution of 10% (v/v) hydrogen peroxide during 1-2 h. Then, spikelets were dehydrated in series of ethanol (50%, 70%, 95% and 100%) and then in a series of solutions with an increasing concentration of methyl salicylate (50%, 75%, 85% and 100%). Cleared ovules were examined in a Leica Diastar 420 DIC microscope (Leica Microsystems).

### Male fertility, female fertility and experimental crossings

Pollen viability was used as an indirect measure of male fertility through the observation of the number of pollen grains stained using an aqueous solution of iodine-potassium iodide (I-KI 1%). The techniques used to collect and stain pollen grains are described in Kearns and Inouye (1993). I-KI molecules stain starch molecules dark blue-violet, pollen grains lacking starch are considered as unviable.

Female fertility was determined by seed set measured in conditions of self-pollination, free-pollination, and experimental cross-pollination. Under self-pollination, spikelets were bagged before anthesis using sulphite paper bags. Under free-pollination, spikelets were bagged after anthesis. Open pollinated seeds obtained from different mother genotypes from population Hojs395 [genotype Hojs395#1 (OP1), and genotype Hojs395#2 (OP2)] and cross-pollinated Hojs395#1 x Hojs395#6 (CP)] seeds were sown and used for progeny test analyses.

For cross-pollination experiments, plants were placed in a fog chamber (Herrmidifier 500, Herrmidifier Parts, OTM Inc., Phoenix, AZ, USA) before anthesis. During early morning when the spikelets start to open, anthers were removed and stigmata were dusted with fresh pollen of selected plants. The procedure was repeated several days. Cross-pollinated flowers were bagged and caryopses were collected after shattering. The number of well-developed and aborted caryopses were separated using a 757 South Dakota Seed Blower (SeedBuro Equipment Company, Des Plaines, IL, USA), and the seeds were stored at 5 °C.

### DNA extractions and fingerprinting

In order to determine the proportion of sexually and apomictically derived offspring, and the sexual potential of tetraploid *P. modestum* genotypes, progeny tests were carried out on 43 individuals obtained from open pollinated and cross-pollinated seeds from three maternal genotypes.

Young leaves were collected in 50 mL polypropylene tubes, immediately frozen in liquid nitrogen (-196 ºC) and stored in a Sanyo VipSeries -86 ºC freezer. The plant material was ground using a porcelain mortar and pestle with liquid nitrogen. DNA was extracted following the methodology by Martínez *et al.* (2003). The extracted DNA was quantified in a SmartSpect 3000 spectrophotometer (Bio-Rad Laboratories, Philadelphia, PA, USA) and the DNA quality was checked in 1% agarose gels stained with ethidium bromide and observed in a UV transilluminator.

RAPD markers were first chosen and 40 arbitrary decamers belonging to the RAPD Primer Synthesis Project of the British Columbia University (UBC series 5 and 8) were tested using the plant material Hojs395 and two progenies, aiming at the selection of most informative primers. However, none of the tested primers showed a clear pattern of molecular bands, and therefore we decided to use AFLP markers instead. For AFLP, we followed the methodology described by Vos *et al.* (1995) with modifications as in Hojsgaard *et al.* (2013). Genomic DNA was digested overnight at 37 °C using 5U of the rare cutter enzyme *Eco*RI (Promega, Madison, WI, USA) and 2.5U of the frequent cutter *Mse*I (New England BioLabs, Ipswich, MA, USA) in a final volume of 25 μL of a 2 Restriction/Ligation (R/L) Buffer [50 mM Tris-HCl, pH 7.5; 50 mM magnesium acetate (C_4_H_6_MgO_4_); 250 mM potassium acetate (C_2_H_3_KO_2_); 25 mM dithiothreitol (DTT)] with 10 mg mL^-1^ of bovine serum albumin (BSA). Reactions were first ice-cold stopped and incubated another 6 h at 37 °C after addition of a ligation mix [5 pM *Eco*RI and 30 pM *Mse*I adapters, 1Weiss U T4 DNA ligase (Promega), 10 mM ATP and 1 R/L buffer]. The pre-selective PCR products were diluted five-folds in ultrapure water, and 5 μL of the dilution was used for the selective amplification using the standard PCR programme. Four out of 20 pre-tested primer pairs were selected for the selective PCR: Mse-AAC-3’/EcoR-AAT-3’, Mse-AAC-3’/EcoR-AGC-3’, Mse/EcoR-AGA-3’, Mse-ACA-3’/EcoR-AAC-3’. The reproducibility of PCRs was checked with three duplicate samples with each primer pair. Amplicons were separated in a 5% (w/v) polyacrylamide denaturing gel using 1 TBE (Tris/Borate/EDTA) buffer at 60 W for 2 h. Gels were fixed in 10% (v/v) acetic acid for 20 min and visualised using the Silver Sequence DNA sequencing system (Promega) according to manufacturer’s recommendations.

### Statistical analyses

A combined binary matrix of the four primer combinations was prepared in Microsoft Office Excel (Microsoft^®^ 2010) and the matrix was assembled into an individual genotype data object with the R package ADEGENET (includes a method that can handle clonal data and allows for analyses of mixed-ploidy data sets with a correction for allele copy-number ambiguity in polyploids) (Jombart, 2008), which was used in the rest of the genetic analyses in the R environment (R Core Team, 2016).

Recombination in all progeny was tested by assessing the genotypic richness, diversity and evenness in the AFLP data set with *poppr* function in the R package *POPPR* (Kamvar *et al.*, 2014). This function calculates the observed Multilocus Genotypes (MLG) and three indices of MLG diversity: Shannon-Wiener index (Shannon, 2001), Stoddart and Taylor’s index (Stoddart and Taylor, 1988), and Simpson’s index (λ) (Simpson, 1949). The hypothesis tested here was that sexual reproduction is observed (non-clonality) in all offspring, thus higher values for the above three indices are expected in progenies compared to a hypothetical clonal progeny (see results). Since MLG is biased against the number of individuals in each progeny, an expected MLG [eMLG - an approximation of the number of expected genotypes at the largest, shared sample size (N=26) based on rarefaction] was calculated and visualized. Similarly, a corrected Simpson’s index was also calculated to avoid the sample size bias using the equation (N/ (N-1)) λ, where N = number of observed samples. Furthermore, the clonality of the members of each progeny was tested using Linkage Disequilibrium. Significant linkage disequilibrium is expected in clonal offspring while it is not expected in sexuals. The hypothesis tested here was that alleles at different loci are not linked and hence recombine freely into new genotypes during the process of sexual reproduction. This was assessed using the standardized index of association, r_d_.

### Ecological data and species distribution modelling

In total, 22 environmental variables were downloaded from open-source databases for niche and ecological assessment of *P. modestum*: 19 bioclimatic variables from WorldClim data set (1950–2000; version 1.4) (Hijmans *et al.*, 2005); http://www.worldclim.org), elevation data of the central South America from the Shuttle Radar Topography Mission (SRTM; http://srtm.csi.cgiar.org/) elevation data set, Photosynthetically available radiation (PAR) data Moderate Resolution Imaging Spectroradiometer (MODIS) database (Myneni *et al.*, 2015); https://lpdaac.usgs.gov), and annual mean UV-B radiation data set from glUV (a global UV-B radiation data set for macro ecological studies) (Beckmann *et al.*, 2014); www.ufz.de/gluv). Data sets with different resolutions were either aggregated or disaggregated to 2.5 arc-min accordingly, using the bilinear method (Hijmans and Van Etten, 2015). Several R packages were used for the preparation of the ecological data for analysis: `sp’ (Bivand *et al.*, 2013) `maptools’ (Bivand and Lewin-Koh, 2013) and `raster’ (Hijmans and Van Etten, 2015).

A raster grid stack of 22 environmental variables (stated above) was generated for South America and the ecological variable values were obtained from point extraction with the collection location geographic coordinates. A principle component analysis was performed on this extracted data to determine the variables that contribute most for the distribution of the species. To avoid overfitting the data, only the variables with a contribution (*CO* values of the PCA) higher than 75% to the first axis of the PCA ordination output were used in the environmental niche assessment and species distribution modelling (see results). Species and distribution models were constructed using MaxEnt v. 3.3.3k (Phillips *et al.*, 2006) and the R package *dismo* (Hijmans *et al.*, 2017). Background data was extracted as pseudo-absence from random points (1300 points) drawn from circular area around the collection points (presence data).

## Results

### Species occurrence and ploidy levels

A total of 137 occurrences were assembled from field observations, herbaria and databases, including 93 obtained from the GBIF database and 35 occurrences from the IRIS database. After removing duplicates and species records without coordinates or appropriate description of the collection point, 43 locality points were retained including all the data from IRIS database and crosschecked to CTES and SI herbaria (Supplementary material Table S1). This information was used in the modelling of species distribution.

**Figure 1:**
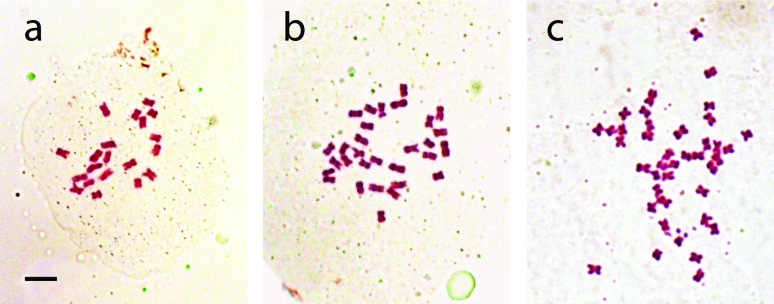
Mitotic metaphases from different *Paspalum modestum* plants. (a) diploid cell with 2*n*=2*x*=20 chromosomes. (b) triploid cell with 2*n*=3*x*=30 chromosomes. c: tetraploid cell with 2*n*=4*x*=40 chromosomes. The scale bar represents 5 μm.

The number of chromosomes of *P. modestum* plants collected from 7 localities (Table 1) was determined after analysing 3-10 metaphase cells (Figure 1). In four localities only diploids (2*n* = 2*x* = 20) were found. A triploid plant (2*n* = 3*x* = 30) was found nearby a crop field in La Leonesa, Chaco (collected by Mario Urbani). In two different collection sites we found both diploid and tetraploid (2*n* = 4*x* = 40) plants growing intermixed (Table 1). Unfortunately, the triploid plant died after ploidy evaluations. Tetraploid individuals from these localities were used for reproductive and genetic evaluations.

### Male meiosis and microsporogenesis

**Figure 2:**
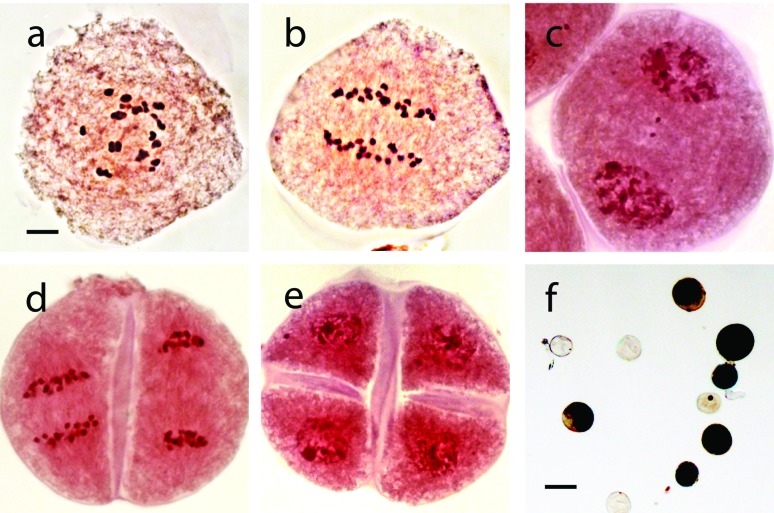
Male gametophyte development in *Paspalum modestum*. (a-e) Progression of male meiosis through different cellular stages: metaphase I (a) showing 1 I + 10 II + 1 III + 4 IV chromosome associations, anaphase I (b), telophase I (c), anaphase II and telophase II (d), and tetrad (e). (f) Mature male gametophytes at blooming. The scale bar in a-e represents 10 μm, and in f represents 100 μm.

Different stages of progression during meiosis and microspore development were analyzed in tetraploid *P. modestum* plants. A total of 92 microspore mother cells were examined, 60 of which were in diakinesis and metaphase I stages of meiosis (Figure 2a). In these stages, chromosomes were associated forming mainly bivalents and tetravalents (Table 2). In anaphase I homologous chromosomes were segregated into two poles (Figure 2b) and 20 single chromatid chromosomes did so during anaphase II (Figure 2c), as expected for a tetraploid plant. In some cells, lagging chromosomes were temporarily observed in the metaphase plate, and were later integrated into respective groups toward the end of anaphase I and beginning of telophase I. In a few cases they remained in the cytoplasm (Figure 2d) and formed micronuclei at tetrads stage (Figure 2e). Microgametogenesis started with the differentiation of the cell wall and a mitotic division to produce a vegetative nucleus and a generative nucleus attached to the inner part of the cell wall. Pollen grains were released mostly as bicellular gametophytes (i.e*.,* before the mitotic division of the generative cell).

**Table 2:** Chromosome configurations found in male meiocytes of tetraploid *Paspalum modestum.*

Plants	Number of PMC	Average and variation () per PMC			
		I	II	III	IV
3	60	1,71 (0–6)	12,78 (5–20)	0,38 (0–4)	2,83 (0–6)

### Male fertility and seed production

A total of 2000 pollen grains from two tetraploid individuals were analyzed. Around 70% of pollen grains were coloured with I-KI (Figure 2f). Meiotic irregularities observed in low frequency might be responsible for the lack of coloration in some pollen grains.

Seed set analysis shows around 70% of spikelets formed caryopses under open pollination (526 spikelets out of 751), 46% under cross-pollination (24 out of 52), and 31% under self-pollination (39 out of 126). These proportions are similar to those found in other apomictic species within *Paspalum*.

### Megasporogenesis, megagametogenesis, and reproductive characterization

The reproductive mode was established through analyses of ovule developmental steps and progeny tests using AFLPs (details in the next section).

**Figure 3:**
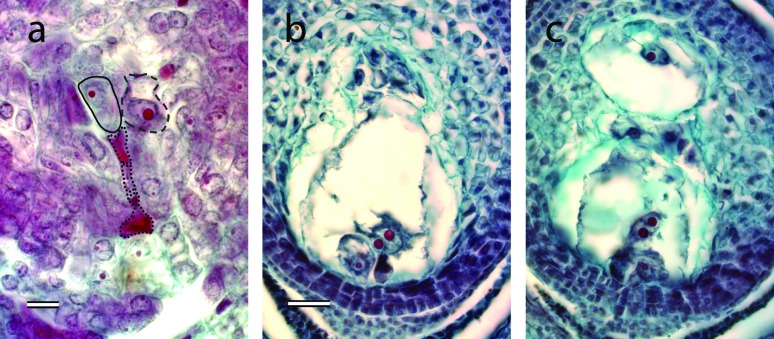
Female gametophyte development in *Paspalum modestum*. (a) Photomicrograph of a section of an ovule at the end of meiosis showing a functional megaspore together with three aborted megaspores and a vacuolated initial of apospory cell. (b) Mature *Polygonum*-type gametophyte depicting an egg-cell, a binucleate central cell and a mass of antipodal cells at the chalazal pole. (c) Mature meiotic gametophyte as shown in b, plus a nucellar gametophyte towards the chalaza. All photomicrographs are disposed with the micropyle towards the base of the figure. The scale bar in a represents 20 μm, and in b-c represents 50 μm.

Megasporogenesis (i.e., meiosis in the archesporial cell) was evaluated in spikelets selected by the presence in anthers of pollen mother cells going through the meiotic division or cells in later stages of microsporogenesis. In ovules of tetraploid plants, the archesporial cell grows from a nucellar cell, differentiates and produces 3 (sometimes 4) megaspores after meiosis, of which only the one located toward the chalaza remained functional (Figure 3a). At this stage, it is possible to observe 1-3(4) nucellar cells surrounding the meiotic products acquiring an intense cytoplasmic coloration and increase in size of their nuclei (Figure 3a). These cells become initials of apospory cells. Both the functional megaspore and the apospory initial cells can develop into a functional female gametophyte. Gametogenesis in the functional megaspore followed the *Polygonum* type development producing an 8-nucleate megagametophyte organized in a 7-celled anatomical structure (Figure 3b). Later, antipodals cells proliferated to produce a mass of cell in variable number, a relevant embryological feature in grasses (Anton and Coccuci, 1984). Gametogenesis in the nucellar cells produced a (3)4(5)-nucleate megagametophyte organized in a (3)4-celled anatomical structure (synergid cells were not always observed) (Figure 3c). Due to the ontogenetic nature of female gametophytes, their structure and genetic constitution was different. The meiotic derived gametophytes had haploid (*n*) nuclei while the apomictic ones had diploid (2*n*) nuclei.

The proportion of mature female gametophytes estimated from spikelets at anthesis is detailed in Table 3. Since aposporous development occurs in a different cell line than the germline, ovules carrying more than one female gametophyte were often observed (Figure 3c, Table 3). Variation in numbers between techniques was likely due to the difficulty of observing antipodals cells using the clearing technique, especially in ovules carrying multiple gametophytes. Hence, the proportion of aposporous embryo sacs can be biased slightly upwards, without affecting the reproductive classification of the material. In general, meiotic embryo sacs were more frequently observed (65% of ovules) compared to AES (44% of ovules).

**Table 3:** Type and numbers of female gametophytes observed in ovules from different tetraploid genotypes of *Paspalum modestum.*

Methodology	Number of ovules	Number of ovules carrying:			
		MES	MES + AES	AES	Ab
Sectioning + staining	43	16	24	2	1
		(32 2%)	(55 8%)	(4 6%)	(2 3%)
Clearing	74	21 (28 3%)	32 (43 2%)	14 (19%)	7 (9 5%)
Total	117 (100%)	37 (31.6%)	56 (47.9%)	16 (13.7%)	8 (6.8%)

### AFLP analysis and test of clonality

Forty-three offspring, including 10 cross-pollinated F_1_ individuals (CP) and 9 and 24 open pollinated individuals (OP1 and OP2) from different genotypes obtained from three mother plants (Hojs395#1, #2, #6) were used in progeny test analysis. In total, 211 AFLP fragments (putative loci) were recovered in the combined data set (an average of 175 loci per individual), of which 42.3% were polymorphic. Different progenies showed dissimilar levels of polymorphism; CP offspring showed 18.2% of polymorphic bands, while OP1 offspring showed 4.70% of polymorphisms and OP2 showed 32.9%. The open pollinated progeny OP1 had the highest number of bands 208, with 18% private fragments. A threshold of > 4 polymorphic bands (a minimum of 5 bands) was considered for classifying an offspring as sexual. Thus, two (20%), one (11.1%), and eight (32%) sexual individuals were observed in CP, OP1, and OP2 offspring, respectively.

**Figure 4:**
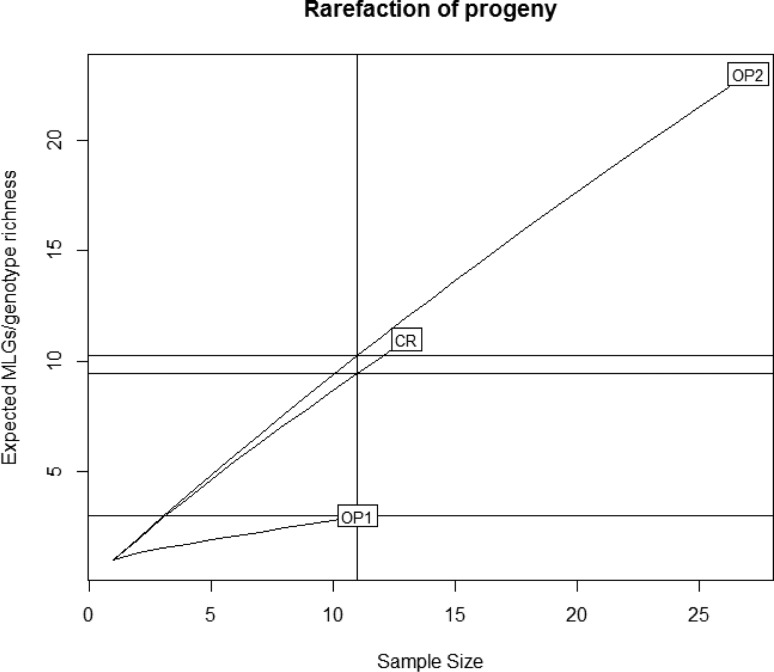
Rarefaction curve for progenies indicating the number of expected multilocus genotypes (eMLGs) together with the minimum number of samples in each progeny. CR: cross pollinated progeny; OP1: open pollinated progeny 1; OP2: open pollinated progeny 2.

The observed number of multilocus genotype (MLGs) was used as a measure of genotypic richness in the progeny with the assumption that sexuals produce higher number of MLGs compared to asexuals (clonals). The lowest MLGs was observed in OP1 (3 MLGs – 27.2%) while the highest was observed in the progeny OP2 (23 MLGs – 85%), indicating higher clonality in the OP1 progeny. After removing the sampling size bias from the analysis (eMLG), the data indicates that progenies CR and OP2 will have higher number of MLG even at the lowest sampling size (11 individuals) (Figure 4). However, only one sexual individual was observed in the OP1 progeny, and since the *poppr* function compares the unique combinations of alleles across two or more loci among individuals in order to estimate MLGs values, we cannot discard a bias in the MLGs richness of the OP1 progeny.

All three diversity indices used in the analysis indicate higher MLG diversity in the OP2 progeny (H - 3.07, G - 19.70, λ - 0.94) (Table 4) suggesting higher sexual recombination. Similarly, diversity indices indicate lowest MLG diversity in the CR progeny (H - 0.60, G - 1.45, λ - 0.31) (Table 4), suggesting higher clonality. When the sample size bias correction was applied to all three progenies, the values slightly changed (see corrected λ in Table 4) without affecting the overall frequencies of clonality. The highest linkage disequilibrium was observed in the progeny OP1 (r_d_ – 0.43) (Figure S1a), likely due to the presence of only 1 recombinant individual and hence, showing significant clonality (*p*-value: 0.001). Although CR and OP2 progenies have lower linkage disequilibrium (0.25 and 0.16 respectively), they also exhibit substantial clonality (Figure S1b,c).

**Table 4:** Analysis of multilocus genotypes diversity in progenies of *Paspalum modestum.*

Progeny	N	MLG	eMLG	SE	H	G	λ	Hexp.	r_d_	Poly (%)	cor. λ	clonal. fr
CR	13	11	9.46	0.57	2.31	8.89	0.89	0.09	0.25	18.82	0.962	0.154
OP1	11	3	3.00	0.00	0.60	1.46	0.31	0.04	0.43	4.70	0.345	0.727
OP2	27	23	10.27	0.74	3.07	19.70	0.95	0.12	0.16	32.94	0.986	0.148
Total	51	36	9.53	1.10	3.32	17.94	0.94	0.20	0.10	42.35%	0.963	0.294

N: Number of individuals observed.

MLG: Number of multilocus genotypes (MLG) observed.

eMLG: The number of expected MLG at the smallest sample size ≥ 10 based on rarefaction

SE: Standard error based on eMLG.

H: Shannon-Wiener Index of MLG diversity (Shannon, 2001).

G: Stoddart and Taylor’s Index of MLG diversity (Stoddart and Taylor, 1988).

λ: Simpson’s Index (Simpson, 1949).

Hexp: Nei’s unbiased gene diversity (Nei, 1978).

rd: The standardized index of association (Agapow and Burt, 2001)

Poly.: percentage of polymorphic markers

cor. λ: Corrected Simpson’s index: equal sample size (0 - no genotypes are different, 1 - all genotypes are different)

clonal.fr: Clonal fraction (0 - no genotypes are clonals, 1 - all genotypes are clonals)

### Ploidy of the sexual offspring

Genetic variable progeny can be obtained through different developmental pathways. In order to properly characterize the reproductive biology of a plant, we need to discriminate between expected B_*II*_ (formed by the fusion of two meiotically reduced gametes) and possible B_*III*_ (involving one apomictically unreduced gamete and one meiotically reduced gamete) progenies. While B_*II*_ individuals from tetraploid plants must have *n* + *n* = 2*x* + 2*x* = 40 chromosomes, a B_*III*_ offspring will have 2*n* + *n* = 4*x* + 2*x* = 60 chromosomes. Among the offspring showing variable genetic markers, chromosome counts revealed no B_*III*_ individuals.

### Species distribution modelling

The contribution values of the PCA of environmental data showed that only 10 out of 22 environmental variables make more than 75% correlation to the distribution of the species: They were five temperature associated variables (Bio1, Bio5, Bio8, Bio10, and Bio11), three precipitation related variables (Bio14, Bio15, and Bio19), and two radiation variables (PAR and UVB) (Figure S2). Therefore, these 10 variables were retained for the species distribution modelling. The MaxEnt model of species distribution performed significantly well for the species distribution prediction with high an AUC (area under the operating curve) value = 0.91, indicating the high accuracy of the model.

**Figure 5:**
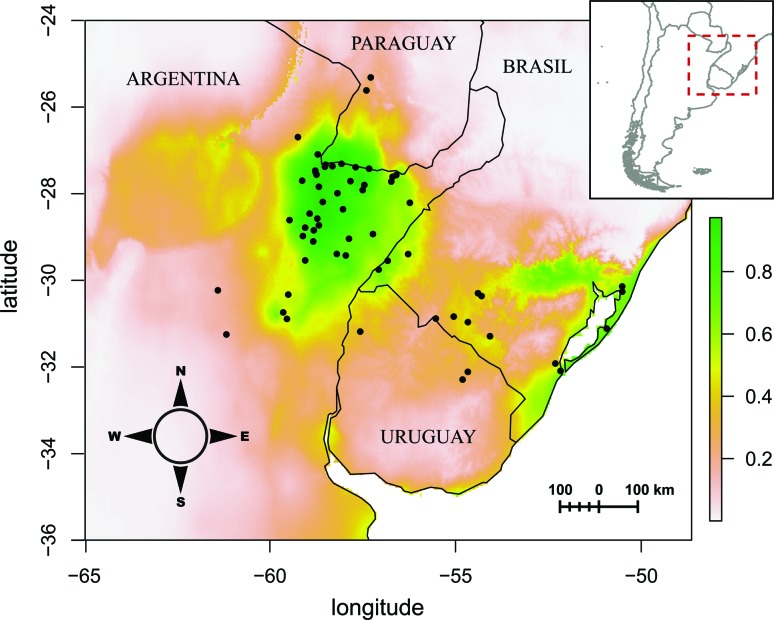
Species distribution model prediction of the probability of occurrence and habitat suitability of *Paspalum modestum* in the studied area and surrounding region. Realized (black dots) and potential habitat suitability (inferred from AUC values) of the species. Green and yellow areas represent the zone where ecological conditions meet the species requirements.

In general, the predicted climatic niche spaces reflect the realized range of distribution of the species, with most localities having prediction scores > 0.65 (strong signals for habitat suitability) (Figure 5). MaxEnt predictions show a close reflection between the environmental niche spaces predicted for the species and its realized niche, having some exceptions in locality points of collections associated to rivers, streams and areas of high anthropogenic impact. For example, the northernmost occurrence point belongs to the Ypacaraí lake in the Department of Cordillera (Paraguay), or the southernmost occurrence to the west of the map is associated to the San Antonio river which is connected to a number of drainage and irrigation channels (Figure 5, Table S1).

To obtain a wider potential climate suitability and distribution ranges, we focused on temperature and precipitation as main drivers and performed the species distribution model with only mean annual temperature (Bio 01) and mean annual precipitation (Bio 12). This model not only produced accurate predictions of current distribution but also showed much wider availability of suitable habitats further north of the currently observed distribution range (see Figure S3).

## Discussion

Biodiversity is a pillar for the maintenance of healthy ecosystems and a central provider of services required for the development of productive societies (Haines-Young and Potschin, 2012). Thus, conservation of biodiversity and sustainable use of native plant resources plays a key role in the well-being of communities. Here we present details on the reproductive biology and ecological preferences of a fodder grass species prone to be exploited as forage adapted to ecosystems with regular floodings and lacking improved forage plants.

### Polyploid origin and reproductive variability in *Paspalum modestum*

Besides the known diploids, new tetraploids and a rare triploid cytotype of *P. modestum* were found, indicating increasing the number and frequency of germplasm collections is central to characterize local plant resources. Ploidy diversity can not only be associated to variable performance and productivity, but also to trait variability, thus having relevant implications in plant breeding (e.g. Zhang *et al.*, 2017).

According to Stebbins (1947), polyploids can be classified following their origin into autopolyploids and allopolyploids. While autopolyploids derive from one parental species and therefore have more than two copies (and sets of homologous chromosomes) of the parental genome, allopolyploids have two parental species and a variable number of copies from each genome. In nature it is possible to find a swarm of polyploid types between auto- and allopolyploids depending upon the genetic differentiation of species. Partially differentiated species are expected to have moderately differentiated genomes and homeologous chromosomes (Soltis *et al.*, 2015 and references therein). Following such genomic similarities, chromosomes will pair to each other during the prophase I of meiosis. In autopolyploid species, the presence of more than two copies of homologous chromosomes will associate and form multivalent structures. In allopolyploid species, chromosomes will form only bivalent associations (nonetheless this will depend upon genetic control and number of genome copies from each parental species; reviewed in Grandont *et al.*, 2013). In *P. modestum*, the frequencies of chromosome associations observed during the first meiotic division in pollen mother cells of tetraploid plants, and particularly the occurrence of up to 6 tetravalents in a single meiotic cell (Table 2), indicates the polyploids have four copies of each (homologous) chromosome and hence, they are expected to be autopolyploids.

Natural populations of *P. modestum* are constituted by diploid, rare triploid, and tetraploid cytotypes, growing separately or together. While the diploids are self-sterile sexuals (Quarin and Hanna, 1980), information on reproductive biology of polyploids was missing. The reproductive analyses of tetraploid plants show a strong variation in the incidence of sexuality and apomixis at different developmental stages. The potential for sex in ovules is almost 80% (75.1-86%; Table 3), but only a small proportion of 26% (11-32%) is realized in the offspring. However, the formation of genetically recombinant progeny does not necessarily imply a fully sexual development. In *Paspalum* species, the formation of sexual progeny usually concurs via a B_*II*_ pathway, but the formation of B_*III*_ individuals is also common (see e.g., Martínez *et al.*, 1994). In spite of the fact that such individuals are developed from a clonal female gamete, fertilization by haploid sperms shifts the ploidy of the offspring and introduces morphological and genetic variability. In our studied plant genotypes, the presence of B_*III*_ offspring is discarded as all sexual classified genetically recombinant progeny have 2*n* = 4*x* = 40 chromosomes, and therefore were formed after the fusion of female and male meiotically reduced gametes carrying 20 chromosomes each. Thus, the observed variation in proportions of sexuality along developmental stages can only be attributed to the efficiency of functional meiosis plus fertilization steps. A depletion of sexuality against apomictic development was already observed in other apomictic species, including monocot species from *Paspalum* spp. (Hojsgaard *et al.*, 2013) and dicot species from *Ranunculus* spp. (Hojsgaard *et al.*, 2014). In *P. modestum*, as well as in the studies mentioned above, the observed decrease of the sexual reproductive efficiency is likely associated to the better competitive ability of apomictic embryo sacs and the high proportion of ovules with both meiotic and apomictic embryo sacs (49.5%; Table 3) in which meiotic development is at disadvantage. The evaluation of male gametophyte development shows no radical changes with respect to other sexual grass species (e.g., Martínez *et al.*, 2007), and the moderately high seed set is supported by the high quality of the pollen produced by tetraploid plants.

### Ecology, sex, apomixis, and breeding potential

Every year, soil flooding affects around 1.700 Mha of land worldwide, including grasslands and pastures grown for livestock (Voesenek and Sasidharan, 2013), and due to climate change floods are expected to increase during the next decades (Hirabayashi *et al.*, 2013). These ecosystems are subjected to drastic seasonal changes that influence plant community composition and vegetation types, holding highly flexible, local adapted plant ecotypes capable to cope with diverse stressors, disturbances, invasion by species from adjacent ecosystems and recolonization (Opperman *et al.*, 2017). Utilizing such ecosystems for forage production requires the selection of adapted species, and appropriate timing of field operations, mainly because these areas can be demanding to establish by conventional seeding methods (Cash *et al.*, 1999). Forage species with high flooding tolerance used in agriculture are few, all from the northern hemisphere or Africa (Striker and Colmer, 2017).

*Paspalum modestum,* is a species adapted to waterlogging that grows in estuaries, borders of shallow waters, and floodplains (Fabbri *et al.*, 2005; Zuloaga and Morrone, 2005). Species distribution modelling depicts a wide geographical area of climatic suitability where *P. modestum* can grow and be exploited as a forage grass. Moreover, the observed occurrences of the species in areas with low habitat suitability scores (i.e., < 0.65 probability; e.g., in Paraguay, central-east Argentina and Uruguay) suggest the species can easily adapt to slightly different environments, not predicted by the distribution modelling. This suggests that the species can exploit a much wider geographic range, provided a minimum number of ecological variables are encountered, likely associated to preferences in temperature range and water regime. A similar dependence of intraspecific variability and species distribution on ecological variables was already found in other *Paspalum* species (Karunarathne *et al.*, 2018). In fact, *P. modestum* distribution modelling focussed on temperature and precipitation as main drivers display much wider potential climate suitability for the species in South America, encompassing previous areas with low habitat suitability scores. Knowledge about the ecological preferences of a species and its potential distribution areas could be used in planning breeding schemes more fitted to specific ecological needs or ecological systems.

Traditional plant breeding focuses on the transfer of characters during sexual reproduction. While sexuality is relevant to segregate quality traits and introgress them into selected genotypes, apomixis is required to fix genetic attributes of superior genotypes. Thus, the occurrence of low levels of sex in predominantly apomictic plants plays a central role in breeding of forage grasses (Hanna and Bashaw, 1987) and proper determination of proportions of sexuality are useful to estimate potential rates of hybridization and time required for character introgression (Hojsgaard *et al.*, 2016). In this sense, tetraploid *P. modestum* genotypes exhibit a good proportion of sexual progeny (26%) compared to other apomictic grasses (e.g. 2.4% in *P. notatum*, Rebozzio *et al.*, 2011; 0.0% in *P. malacophyllum*, Hojsgaard *et al.*, 2013) that can be easily used to transfer and/or select superior features. We know that the species is cross-compatible with the forage grass *P. notatum* (Quarin, 1983) and likely to other relevant forage grasses within the close related group of Plicatula species (e.g., *P. plicatulum*). First, selected *P. modestum* genotypes can thereby be used as maternal parents to be pollinated by other intraspecific or interspecific apomicts to produce new gene combinations or experimental hybrids. Later, maximizing the expression of apomixis is crucial to maintain phenotypic homogeneity needed for developing commercial varieties. On the one hand, interspecific hybrids had already been produced for several species of Plicatula, including species widely used in breeding programs like *P. plicatulum* and *P. guenoarum* (Aguilera *et al.*, 2011), and species not used yet in breeding programs, but with similar ecological preferences to those of *P. modestum* like *P. wrightii* Hitchc. & Chase (sub *P. hydrophilum* Henrard) and *P. palustre* Mez (Martínez and Quarin, 1999). On the other hand, in plants, reproductive biology (e.g., flowering time) is influenced by climatic fluctuations with important effects on plant performance (Hampton *et al.*, 2016), and in apomictic plants environmental changes also affect the expression of sexuality and apomixis (e.g., Klatt *et al.*, 2016). Hence, knowing the penetrance of apomixis and the ecological spectrum in which a plant ecotype can grow contribute relevant information on regional possibilities to maximize apomixis in forage fields minimizing formation of non-maternal offspring.

Thus, considering the particular ecosystem in which the species grow (Zuloaga and Morrone, 2005), the ecological and reproductive flexibility the species exhibit, their attributes as forage grass (Reis, 2007) and crossability to other forage species, *P. modestum* can be considered a native plant resource with very good potential for breeding different ecotypes and genotypes and developing cultivars of high biomass production and waterlogging tolerances adapted to low flow areas in the Subtropics of Brazil, Paraguay, Uruguay and Argentina.

### Conclusion and future prospects

Forage breeding is central for intensification of cattle production. The use of native plant genetic resources can benefit from genotypes better adapted to local conditions and from avoiding negative impacts of plant introductions. Understanding the ecological potential of a species with forage aptitudes is recommendable to predict suitable areas of cultivation and zones in which the performance of plant material can be maximized. *Paspalum modestum* is a grass species that combines facultative apomictic polyploids, good forage aptitudes, and is adapted to ecosystems poorly exploited with cultivated pastures, yet with high potential for growing stocking rates. Increasing the number of plant collections and characterization of regional germplasm of *P. modestum* materials will facilitate its inclusion in breeding plans.

## Supplementary material

The following online material is available for this study

Figure S1 Linkage disequilibrium plots of progenies.

Figure S2 PCA indicating the contribution of each environmental variable for the observed distribution of *Paspalum modestum*.

Figure S3 Species distribution model prediction.

Table S1 List of *Paspalum modestum* localities used for ecological niche modelling.
