# Supplementary material for: Ecological setup, ploidy diversity, and reproductive biology of Paspalum modestum, a promising wetland forage grass from South America
Source: Genet Mol Biol. 2020 Feb 21;43(1 Suppl 2):e20190101. doi: 10.1590/1678-4685-GMB-2019-0101 (PMC7198000; doi:10.1590/1678-4685-GMB-2019-0101)
Supplement: Supplementary file 2 [file 1415-4757-GMB-43-1-s2-e20190101-s1.pdf]

Genetics and Molecular Biology 43,1(suppl 2) (2020)

Supplementary Material to "Ecological setup, ploidy diversity, and reproductive biology of *Paspalum modestum*, a promising wetland forage grass from South America"

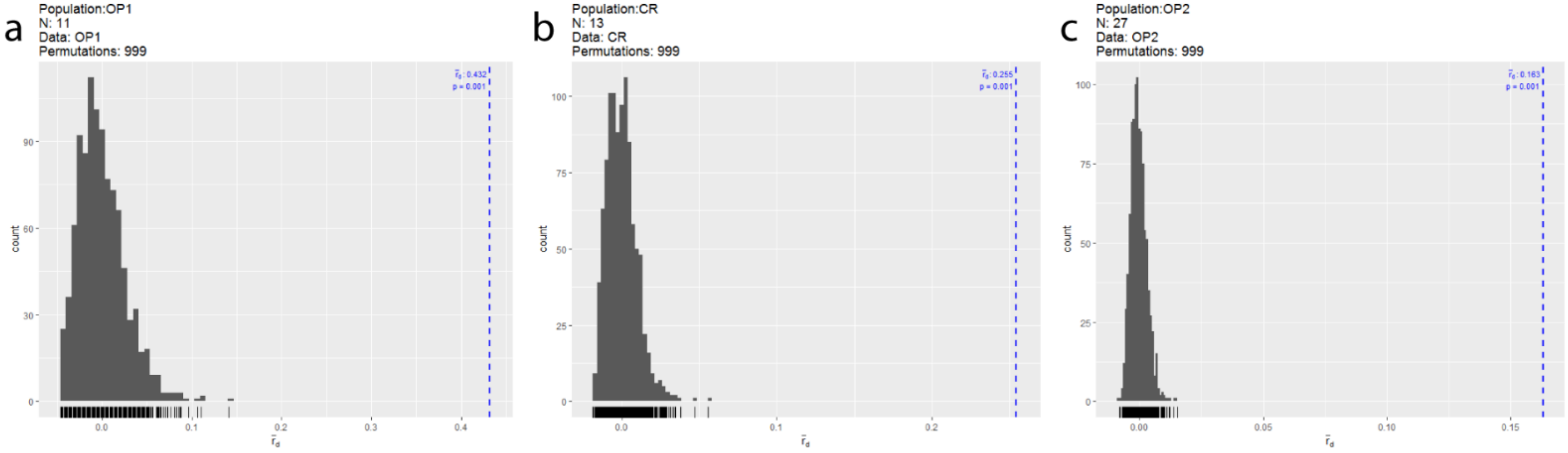

Figure S1 - Linkage disequilibrium plots of progenies. **a:** open pollinated progeny 1. **b:** CR: cross pollinated progeny. **c:** open pollinated progeny 2
