# Supplementary material for: Ecological setup, ploidy diversity, and reproductive biology of Paspalum modestum, a promising wetland forage grass from South America
Source: Genet Mol Biol. 2020 Feb 21;43(1 Suppl 2):e20190101. doi: 10.1590/1678-4685-GMB-2019-0101 (PMC7198000; doi:10.1590/1678-4685-GMB-2019-0101)
Supplement: Supplementary file 4 [file 1415-4757-GMB-43-1-s2-e20190101-s3.pdf]

Supplementary Material to "Ecological setup, ploidy diversity, and reproductive biology of *Paspalum modestum*, a promising wetland forage grass from South America"

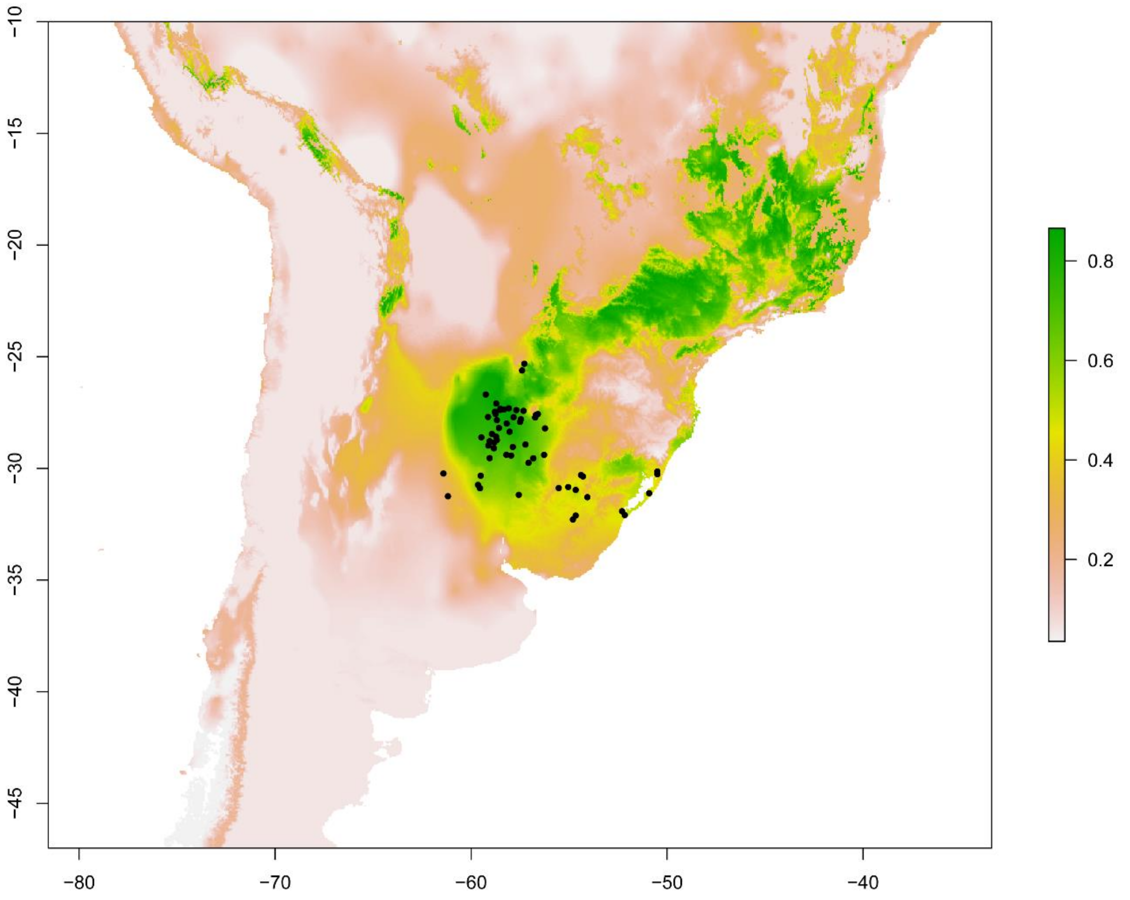

Figure S3 - Species distribution model prediction using mean annual temperature (Bio 01) and mean annual precipitation (Bio 12) variables. Habitat suitability of the species (inferred from AUC values) showing the zone where ecological conditions meet the requirements of the species (green and yellow shadows).
